# Effects of dietary supplementation of nickel and nickel-zinc on femoral bone structure in rabbits

**DOI:** 10.1186/1751-0147-51-52

**Published:** 2009-12-15

**Authors:** Monika Martiniaková, Radoslav Omelka, Birgit Grosskopf, Hana Chovancová, Peter Massányi, Peter Chrenek

**Affiliations:** 1 Department of Zoology and Anthropology, Constantine the Philosopher University, Nábrežie mládeže 91, 949 74 Nitra, Slovak Republic; 2 Department of Botany and Genetics, Constantine the Philosopher University, Nábrežie mládeže 91, 949 74 Nitra, Slovak Republic; 3 Johann Friedrich Blumenbach Institute of Zoology and Anthropology, Georg-August University, Bürgerstrasse 50, 37 073 Göttingen, Germany; 4 Department of Animal Physiology, Slovak University of Agriculture, Trieda A. Hlinku 2, 949 76 Nitra, Slovak Republic; 5 Slovak Agricultural Research Centre, Hlohovská 2, 949 92 Nitra, Slovak Republic

## Abstract

**Background:**

Nickel (Ni) and zinc (Zn) are trace elements present at low concentrations in agroecosystems. Nickel, however, may have toxic effects on living organisms and is often considered as a contaminant. This study reports the effect of peroral administrated Ni or a combination of Ni and Zn on femoral bone structure in rabbits.

**Methods:**

One month-old female rabbits were divided into three groups of five animals each. Group 1 rabbits were fed a granular feed mixture with addition of 35 g NiCl_2 _per 100 kg of mixture for 90 days. In group 2, animals were fed a mixture containing 35 g NiCl_2 _and 30 g ZnCl_2 _per 100 kg of mixture. Group 3 without administration of additional Ni or Zn served as control. After the 90-day experimental period, femoral length, femoral weight and histological structure of the femur were analyzed and compared.

**Results:**

The results did not indicate a statistically significant difference in either femoral length or weight between the two experimental groups and the control group. Also, differences in qualitative histological characteristics of the femora among rabbits from the three groups were absent, except for a fewer number of secondary osteons found in the animals of groups 1 and 2. However, values for vascular canal parameters of primary osteons were significantly lower in group 1 than in the control one. Peroral administration of a combination of Ni and Zn (group 2) led to a significant decreased size of the secondary osteons.

**Conclusions:**

The study indicates that dietary supplementation of Ni (35 g NiCl_2 _per 100 kg of feed mixture) and Ni-Zn combination (35 g NiCl_2 _and 30 g ZnCl_2 _per 100 kg of the mixture) affects the microstructure of compact bone tissue in young rabbits.

## Background

Pollution of the environment and exposure of humans and animals to trace elements represent serious problems in many countries. The amount of an element, which accumulates in various tissues including bone, depends on the period of exposure, the exposure level, absorption, the production and reproduction phases of the individual, as well as species, age and breed [1]. Trace element toxicity upon the biological systems of animals is influenced by the route and form of exposure as well as by the interaction between beneficial and toxic substances [2]. Some elements are essential for life, others have unknown biological functions and some may have toxic effects and be associated with development of disease [3,4].

In general, nickel (Ni) is a widely distributed metal that is industrially applied in many forms. It is an essential mineral that may accumulate to high levels in soil and consequently contaminated soil may be a source for potential intoxication [5]. Drinking water and food are the main sources of exposure for the general population. Ni compounds are carcinogenic to humans and are potent inducers of kidney and lung tumors in experimental animals [6]. Reduced growth rate, reduced reproductive rates, and alterations of serum lipids and glucose have been observed in animal studies [7]. Ni intoxication is associated with lesions and dysfunction of the testes, seminal vesicle, and prostate gland [8-10]. According to Chen *et al*. [11], Ni may have an oxidative effect on bone marrow. However, its effect on compact bone microstructure has not been investigated yet.

Zinc (Zn) is an essential trace element which stimulates the activity of many enzymes. Among other effects, Zn supports the immune system [12], is needed for wound healing [13] and for DNA synthesis [14]. Zn is also believed to have specific anti-atherogenic properties by inhibiting oxidative stress-responsive transcription factors that are activated during an inflammatory response [15]. The study by Fabris and Mocchegiani [16] suggests that Zn supports fetal and post natal growth and development. Zn also plays an important role in bone metabolism [17].

In vitro studies suggest that Ni and Zn behave similar at certain sites in biological systems. Ni and Zn are consistently found in high concentrations together with RNA and DNA and may stabilize their structure. Ni and Zn also contribute to ribosomal conformation [18] and serve as enzyme activators, i.g., arginase can be activated by either Zn or Ni [19]. Finally, the white blood cell is a possible site of interaction between the two elements [18] as both elements increase the adhesiveness of polymorphonuclear leukocytes [20]. Antagonistic effects of Zn towards Ni toxicity have been observed as Zn is effective in alleviating the Ni induced toxicity in the liver of rats [21].

The present study investigates the effects of Ni (as NiCl_2_) and a combination of Ni and Zn (as NiCl_2 _and ZnCl_2_) on rabbit femoral bone structure following peroral administration.

## Methods

### Animals

Fifteen clinically healthy 1 month-old female rabbits (*Oryctolagus cuniculus*, Californian breed, broiler line) were divided into three groups of five animals each. The animals (weighting approx. 3.65 - 3.95 kg) were obtained from an experimental farm of the Slovak Agricultural Research Centre (SARC), Nitra, Slovakia. They were housed in individual flat-deck wire cages (area 0.34 m^2^) under a constant photoperiod of 14 h of day-light. Daily physical activity was restricted by the cage size. The temperature (18-20°C) and humidity (65%) of the building were recorded continually by means of a thermograph positioned at the same level as the cages.

Group 1 rabbits were fed a granular feed mixture with the addition of 35 g NiCl_2 _per 100 kg of mixture for 90 days. In the second group, animals were fed a mixture containing 35 g NiCl_2 _and 30 g ZnCl_2 _per 100 kg mixture. Group 3 which received a diet without Ni or Zn added served as control. The rabbits were kept for other investigations (e.g., histological analyses, cytogenetic analyses of blood cells, and biochemical analyses) at SARC. The present study was performed as an additional investigation. Animals were weighted weekly and weight changes were recorded. All procedures were approved by the Animal Experimental Committee of the Slovak Republic.

### Procedures

The animals were euthanized by electrocution after an experimental period of 90 days and bone specimens were sampled during necropsy. Before histological analysis, all femora (n = 30) were measured and weighted by a sliding instrument and analytical scales. Only the right femur was used for histological analysis. The bones were sectioned at the diaphyseal midshaft thus providing a large area of compact bone for study. Two transversal sections were taken of each femur. The isolated segments were macerated and degreased, followed by embedding in epoxy resin (Biodur, Günter von Hagens, Heidelberg, Germany). Histological sections (70-80 μm thick) were prepared with a sawing microtome (Leitz 1600, Leica, Wetzlar, Germany) and affixed to glass slides by Eukitt (Merck, Darmstadt, Germany) as previously described [22].

The qualitative histological characteristics were determined according to the classification systems by Enlow and Brown [23] and Ricqlès *et al. *[24]. The quantitative parameters were assessed using the software Scion Image (Scion Corporation, Maryland, USA) in anterior, posterior, medial and lateral views.

Since osteon size may vary in the different views [25], we measured area, perimeter, and the minimum and maximum diameter of primary osteons' vascular canals, Haversian canals and secondary osteons in all views in order to minimize inter-animal differences. At least 20 vascular canals of primary osteons were measured in each individual (5 each of anterior, posterior, medial and lateral views). All secondary osteons, which were not in a resorption phase and could clearly be outlined using the Scion Image software, were measured. Magnification of 200 times was used for histomorphometric measurements. Before measuring, the sections were carbonized [26]. With this method older osteons achieve a brighter color than young osteons and reversal lines of secondary osteons become much more visible. Secondary osteons were distinguished from primary osteons (i.e., primary vascular canals) on the basis of the well defined peripheral boundary (cement line) between the osteon and the surrounding tissue.

### Statistics

The analysis of variance and Tukey's test (Statistica 4.3, 1993) were used to analyze data on bone length, bone weight, and the quantitative histological characteristics.

## Results

### Gross analysis of bones

Growth rate was similar for all groups (Table 1). Analyses of data on femoral length and femoral weight demonstrated no significant differences. The highest values for the bone length and bone weight were recorded in rabbits from the group fed the diet with a combination of Ni and Zn (group 2) followed by rabbits from the control group and those from group 1. A statistically significant difference was observed only for femoral weight between the two experimental groups (Table 2).

**Table 1:** Body weight

Day	0	7	14	21	28	35	42	49	56	63	70	77	84	90
Group 1	3.73	3.89	4.04	4.11	4.14	4.19	4.27	4.28	4.34	4.10	4.06	4.11	4.01	3.97

Group 2	3.95	4.08	4.29	4.35	4.42	4.53	4.61	4.67	4.81	4.59	4.61	4.71	4.62	4.61

Group 3	3.65	3.80	3.95	3.97	4.00	4.05	4.08	4.08	4.14	4.05	4.02	4.14	3.97	3.98

Differences were not significant (*P *> 0.05)

Average development in body weight (kg) in 3 groups of rabbits (n = 5 per group) receiving either 35 g NiCl_2 _per 100 kg of mixture (group 1), 35 g NiCl_2 _and 30 g ZnCl_2 _per 100 kg of mixture (group 2) or no additional Ni or Zn (group 3).

**Table 2:** Femoral length and weight

Group	No of bones	Femoral length (cm)	Femoral weight (g)
1	10	10.52 ± 0.27	15.78 ± 1.42

2	10	10.78 ± 0.29	17.76 ± 2.05

3	10	10.63 ± 0.37	16.13 ± 1.07

Tukey's test*			1 *vs *.2 +

*P *< 0.05 (+)

* Numbers (1-3) refer to group numbers

Average femoral length and weight in 3 groups of rabbits (n = 5 per group) receiving either 35 g NiCl_2 _per 100 kg of mixture (group 1), 35 g NiCl_2 _and 30 g ZnCl_2 _per 100 kg of mixture (group 2) or no additional Ni or Zn (group 3) for 90 days.

### Qualitative histological characteristics

All animals had a common bone microstructure characterized by the presence of a primary vascular longitudinal bone tissue forming the periosteal surface. This type of the bone tissue was created by vascular canals which ran in a direction essentially parallel to the long axis of the bone. In middle parts of the compact bone, a dense Haversian bone tissue and/or a primary vascular radial bone tissue was present. Dense Haversian bone tissue was due to a large number of secondary osteons. Primary vascular radial bone tissue was composed of branching or nonbranching vascular canals radiating from the marrow cavity or periosteum and partially extending across the compacta. Endosteal border consisted of a layer of dense Haversian bone tissue.

Significant differences in qualitative histological characteristics of the compact bone tissue were not found although fewer secondary osteons were found in animals from the experimental groups.

### Quantitative histological characteristics

Data on bone measurements are shown in tables 3, 4 and 5. The values for the vascular canals of primary osteons were the highest in group 2 rabbits and lowest in group 1 animals. Significant differences were found between groups 1 and 2 and between group 1 and the control group. The minimal diameter of primary osteons' vascular canals was also significant different between group 2 and the control group. The highest values for all parameters of the Haversian canals were recorded in group 1 rabbits but for Haversian canals, significant differences were not found. All parameters of the secondary osteons had the highest values in rabbits administered only Ni (group 1). However, the parameters were not significantly higher than those of the control group. The lowest values of most parameters of the secondary osteons were observed in rabbits of group 2. Statistically significant differences were identified for perimeter (*P *< 0.05) and maximum diameter (*P *< 0.01) of the secondary osteons between groups 2 and 3.

**Table 3:** Data on vascular canals of primary osteons

Group	No of sections	Area (μm^2^)	Perimeter (μm)	Max. diameter (μm)	Min. diameter (μm)
1	196	190.93 ± 100.82	40.91 ± 9.83	18.59 ± 5.67	6.38 ± 1.86

2	222	253.74 ± 80.32	46.98 ± 7.27	21.27 ± 4.66	7.63 ± 1.84

3	204	235.24 ± 83.81	45.74 ± 8.09	20.98 ± 4.95	7.09 ± 1.67

Tukey's test*		1 *vs *.2 & 3 ++	1 *vs *.2 & 3 ++	1 *vs *.2 & 3 ++	1 *vs *.2 & 3 ++
		
					2 *vs *.3 ++

*P *< 0.01 (++)

* Numbers (1-3) refer to group numbers

Data on vascular canals of primary osteons in 3 groups of rabbits (n = 5 per group) receiving either 35 g NiCl_2 _per 100 kg of mixture (group 1), 35 g NiCl_2 _and 30 g ZnCl_2 _per 100 kg of mixture (group 2) or no additional Ni or Zn (group 3) for 90 days.

**Table 4:** Data on Haversian canals

Group	No of sections	Area (μm^2^)	Perimeter (μm)	Max. diameter (μm)	Min. diameter (μm)
1	66	373.08 ± 173.55	55.96 ± 11.86	25.50 ± 6.64	9.13 ± 2.47

2	87	335.54 ± 115.93	53.08 ± 8.61	23.94 ± 5.04	8.89 ± 2.16

3	105	344.70 ± 154.51	54.02 ± 11.20	24.54 ± 6.39	8.78 ± 2.19

Tukey's test*		NS	NS	NS	NS

* Numbers (1-3) refer to group numbers, NS -- non-significant changes

Data on Haversian canals in 3 groups of rabbits (n = 5 per group) receiving either 35 g NiCl_2 _per 100 kg of mixture (group 1), 35 g NiCl_2 _and 30 g ZnCl_2 _per 100 kg of mixture (group 2) or no additional Ni or Zn (group 3) for 90 days.

**Table 5:** Data on secondary osteons

Group	No of sections	Area (μm^2^)	Perimeter (μm)	Max. diameter (μm)	Min. diameter (μm)
1	66	10034.81 ± 3486.18	284.10 ± 53.22	134.01 ± 28.82	46.75 ± 9.75

2	87	8601.44 ± 2671.20	258.69 ± 42.36	118.15 ± 25.83	46.33 ± 8.57

3	105	9580.88 ± 3344.45	278.93 ± 55.20	132.83 ± 33.44	45.44 ± 8.50

Tukey's test*		1 *vs *.2 +	1 *vs *.2 +	1 *vs *.2 ++	
		
			2 *vs *.3+	2 *vs *.3 ++	

*P *< 0.05 (+), P < 0.01 (++)

* Numbers (1-3) refer to group numbers

Data on secondary osteons in 3 groups of rabbits (n = 5 per group) receiving either 35 g NiCl_2 _per 100 kg of mixture (group 1), 35 g NiCl_2 _and 30 g ZnCl_2 _per 100 kg of mixture (group 2) or no additional Ni or Zn (group 3) for 90 days.

## Discussion

To date, only a few studies focusing on the effects of Ni and Zn supplementation on body weight or size of anatomical structures have been published. Pandey *et al. *[27] found no change in body weight of mice orally administered by nickel sulphate. However, significant decreases in absolute and relative weight ratios of testes, seminal vesicles and prostate gland were seen. On the other hand, Bersényi *et al. *[28] found that supplementation of the diet with 50 mg Ni (as NiCl_2_) per kg of feed was not associated with a significant increase of body weight in rabbits. Similar to this, we did not observe a significant effect on weight or length of the femur in rabbits fed the diet with only Ni added (group 1) when compared to the control group, thus indicating that peroral administration of Ni at the levels used in this study does not influence bone growth in young rabbits significantly. It was also observed that use of a Ni-Zn supplemented diet did not influence bone weight significantly when compared to the controls (Group 2 *vs. *3). However, when the two experimental groups were compared, a significant higher bone weight occurred in the Ni-Zn supplemented group. It is generally known that deficiency of Zn in the body may lead to damage of bone tissue [29]. In addition, Zn plays a physiological role in bone growth by stimulating protein synthesis [30]. Therefore, it is not surprising that the highest values of femoral length and weight have been identified in rabbits from the second group.

The qualitative histological results correspond with those reported by others [22,31,32]. The basic structural pattern of compact bone tissue was primary vascular longitudinal in all groups. In addition, dense Haversian bone tissue and/or primary vascular radial bone tissue were found in the middle part of the compacta. However, a decreased number of secondary osteons was observed in rabbits of groups 1 and 2. It has been shown that circumferential lamellae are substituted by the secondary osteons with increased age in mice [33] and rabbits [22]. Besides that, the formation of secondary osteons tends to lead to the production of more secondary osteons during the life of the individual [32]. Bone remodeling (i.e., formation of new secondary osteons) was most likely reduced in rabbits of groups 1 and 2, probably due to accumulation of Ni in the bones. Nickel accumulates in the kidneys, bones, heart and liver of rabbits following dietary supplementation of 50 and 500 mg Ni (as NiCl_2_) per kg of feed [28].

The quantitative histological analyses showed that values for vascular canal parameters of primary osteons were significantly lower in group 1 rabbits than in control animals. Peroral administration of the Ni-Zn combination (group 2) was associated with a significant decreased size of the secondary osteons. The presence of statistically significant differences in most parameters of primary osteons' vascular canals and secondary osteons in rabbits from the experimental groups (1 and 2) could indicate a protective potential of Zn to Ni induced changes in compact bone microstructure.

This study seems to be the first on the effects of peroral Ni and Ni-Zn supplementation on the microstructure of rabbit compact bone. Comparison with other studies was therefore not possible. Additional research dealing with the influence of trace elements on compact bone structure is required to gain more information and to verify the results of this study. One aim of our current research is to obtain detailed information on trace element associated mechanisms in the development of compact bone and to understand the interactions of different trace elements in compact bone development.

## Conclusions

The study demonstrated that peroral administration of Ni (35 g NiCl_2 _per 100 kg of feed mixture) or supplementation with a combination of Ni and Zn (35 g NiCl_2 _and 30 g ZnCl_2 _per 100 kg of the mixture) affects the microstructure of compact bone tissue in young rabbits following a study period of 90 days. Higher content of Ni significantly decreased the size of the primary osteons' vascular canals. Peroral administration to rabbits of the Ni-Zn supplemented diet led to a significantly decreased size of the secondary osteons. The study seems to be the first dealing with compact bone microstructure in rabbits after peroral administration of Ni or a Ni-Zn combination. The results can be applied in experimental studies focusing on the effects of various trace elements on bone structure.

## Competing interests

The authors declare that they have no competing interests.

## Authors' contributions

MM carried out the histological analysis of examined bones. RO performed the statistical analysis of presented data. BG prepared thin sections for histological analysis. HC carried out macroscopical analysis of femora and photodocumentation of thin sections. PM monitored the food intake of rabbits. PC supported animal care and sampling of femora. All authors read and approved the final manuscript.
